# Examining the effect of Early Life Stress on autonomic and endocrine indicators of individual stress reactivity

**DOI:** 10.1016/j.ynstr.2018.100142

**Published:** 2018-12-17

**Authors:** Luisa Bönke, Sabine Aust, Yan Fan, Katharina Wirth, Elissa Khawli, Amie Stevense, Ana Herrera, Andrea Loayza, Malek Bajbouj, Simone Grimm

**Affiliations:** a Charité – Universitätsmedizin Berlin, Corporate Member of Freie Universität Berlin, Humboldt - Universität zu Berlin, And Berlin Institute of Health, Campus Benjamin Franklin, Hindenburgdamm 30, 12203, Berlin, Germany; b Department of Psychiatry, Psychotherapy and Psychosomatics, Psychiatric Hospital, University of Zurich, Lenggstrasse 31, 8032, Zurich, Switzerland; c Department of Psychology, MSB Medical School Berlin, Calandrellistr. 1-9, 12247, Berlin, Germany

**Keywords:** Early life stress, Stress reactivity, Heart rate, Salivary cortisol level

## Abstract

Early life stress (ELS) is associated with altered stress reactivity and an increased risk for the development of psychopathological conditions in later life. However, depending on whether autonomic or endocrine measures were used as indicators of stress reactivity, previous studies reported conflicting findings of either increased or decreased stress reactivity after ELS experience. In the present study we therefore aimed to investigate the effect of ELS on both autonomic and endocrine indicators (heart rate and salivary cortisol) of individual stress reactivity and applied a psychosocial stress task in a sample of healthy participants with and without exposure to mild to moderate ELS. Results showed no significant effects of ELS on autonomic and endocrine indicators of individual stress reactivity. Importantly though, heart rate proved as more sensitive than salivary cortisol with regard to differentiating between stress and control conditions and thereby as a more feasible indicator of an individual's stress reactivity. Accordingly, our data suggest that sole reliance on salivary cortisol as an indicator of stress reactivity might lead to an oversight of more subtle effects of psychosocial stress.

## 1 Background

The experience of stress initiates important psychological and physiological changes, which serve to provide energy to protect the individual from the potential threat caused by the stressor and thus facilitate adaptation to the stressful situation (Loman and Gunnar, 2010; Zautra, 2003). The hypothalamic-pituitary-adrenal (HPA) axis, indexed by increased levels of glucocorticoids like cortisol, and the sympathetic-adrenal-medullary (SAM) system, indexed by increased cardiovascular activity, are involved especially in stress reactivity (Lovallo, 1997).

In order to allow effective coping with threatening situations, the stress system regulates itself through complex negative feedback mechanisms. Yet it is important to note that under certain circumstances these feedback mechanisms and consequently the stress reactivity may be altered (Zautra, 2003). Both excessive and dampened reactivity of the stress system have been associated with increased vulnerability for psychiatric disorders (Carpenter et al., 2009).

Considering this, the question arises how differences in stress reactivity emerge. Proposed influences are genetic as well as environmental factors (Boyce and Ellis, 2005), with Early Life Stress being one of the most potent of these.

The term Early Life Stress (ELS) subsumes childhood adversities including various kinds of maltreatment and household dysfunctions. Confrontation with these adversities can exceed the child's coping resources and thus lead to persistent phases of stress (Pechtel and Pizzagalli, 2011). In 2013 the National Child Abuse and Neglect Data System (NCANDS; U.S. Department of Health and Human Services) reported 678,932 victims of child maltreatment, leading to a victim rate of 9.1 in 1000 children. Child maltreatment can be defined as “any act or series of acts of commission or omission by a parent or other caregiver that results in harm, potential for harm, or threat of harm to a child” (Leeb et al., 2008, p. 11). *Acts of commission* include physical abuse, sexual abuse and psychological (emotional) abuse whereas *Acts of omission* include physical neglect, emotional neglect, medical/dental neglect and educational neglect as well as inadequate supervision and exposure to violence (Leeb et al., 2008). ELS may lead to physical, cognitive, and psychopathological impairments such as higher risk for cardiovascular disease, depression, and anxiety disorder as well as impaired cognitive performance (Enoch, 2011; Fagundes and Way, 2014; Fuge et al., 2014; McLaughlin et al., 2010; Pechtel and Pizzagalli, 2011; Schilling et al., 2008; Shonkoff et al., 2012).

Considering that, per definition, ELS implies persistent phases of stress, both animal and human studies have investigated the association between ELS and stress reactivity. Yet, the findings remain contradictory, with some studies suggesting that ELS is associated with diminished adrenocorticotrophic hormone (ACTH), cortisol, and heart rate responses to stress tests in adulthood whereas others report an association with an increased stress reactivity, thus increased ACTH, cortisol and heart rate responses (Carpenter et al., 2007; Heim and Nemeroff, 1999, 2002; Lovallo et al., 2012). Partly these differing findings might be due to methodological aspects: There is a great variety concerning the assessment of ELS as well as variety concerning the type of adversity, age at onset, its frequency, severity, and chronicity. Furthermore the utilized laboratory stressors as well as indicators of stress reactivity vary across studies (Heim and Nemeroff, 2001). The gold standard for inducing psychosocial stress in a laboratory setting is the Trier Social Stress Test (TSST, Kirschbaum et al., 1993), where subjects are asked to give a speech and perform a mental arithmetic task in front of an audience. The TSST is associated with 2- to 4-fold elevated salivary cortisol level as well as a significantly increased heart rate in healthy subjects.

Apart from these methodological aspects, there is another important aspect to take into account. The conflicting findings regarding the effects of ELS on stress reactivity might also be due to the fact that the impact of ELS on autonomic and endocrine functioning might differ (Elzinga et al., 2008; Heim et al., 2000). Accordingly, Elzinga et al. (2008) utilized the TSST and reported increased cortisol reactivity, but no changes in cardiovascular reactivity in participants with ELS experience. Thus, some elements of the stress system might be more sensitive to ELS effects than others. As the SAM-system, which includes cardiovascular reactivity, is responsible for short-term reactions whereas the HPA-axis is activated during prolongued stressful situations, it could be assumed that ELS has a specific effect on the functioning of the HPA-axis and thus the cortisol reactivity (Elzinga et al., 2008).

Considering the potentially differing impact of ELS on endocrine and autonomic functioning, it is important to note, that previous research has strongly focused on salivary cortisol as sole indicator of stress reactivity (Gunnar et al., 2009; Lovallo et al., 2012). Due to this common practice, several problems emerge. First of all, cortisol data are influenced by various factors such as circadian rhythm, eating, smoking, physical activity, gender, or menstrual cycle (Dickerson and Kemeny, 2004; Kirschbaum et al., 1993). Moreover, cortisol peaks are generally reached around 20 min after stressor onset with considerable fluctuation concerning this baseline to peak interval (Dickerson and Kemeny, 2004). Accordingly, cortisol might not be an appropriate indicator of stress reactivity in all settings, e.g. for the Montreal Imaging Stress Task (MIST, Dedovic et al., 2005), which is the most commonly used task for inducing psychosocial stress in fMRI settings. During the MIST, subjects perform mental arithmetic tasks in numerous runs of several minutes duration, with each run containing three conditions (rest, control, and stress condition). Consequently, there is a relatively quick change in runs and conditions, suggesting that cortisol might not be suited to differentiate effectively between these (for more detailed description of the MIST see below). Additionally, collecting saliva samples in the fMRI environment tends to be difficult as participants are not supposed to move their head and mouth and accordingly, the collected amount of saliva is often not sufficient for analyzing cortisol concentrations. Moreover, usually only 50% of the participants show a significant cortisol reaction in this task, thus leading to a differentiation between responders and non-responders (Dedovic et al., 2009; Pruessner et al., 2008), while at the same time there is no common standard of how responders and non-responders are defined (Miller et al., 2013). Often, reported results refer to the cortisol responder group only (e.g. Dedovic et al., 2005), which implies that 50% of the collected data are not being used for analysis.

To sum up, even though previous research has strongly focused on salivary cortisol as an indicator of stress reactivity, serious issues concerning the collection and analysis of salivary cortisol data exist. Moreover, the effect of ELS on stress reactivity generally seems to be unclear. To further explore the effect of ELS on stress reactivity, the current study aims to examine the effect of ELS on heart rate and salivary cortisol level as distinct indicators of the individual stress reactivity, inducing stress with the MIST. This will allow us to not only gain further insight into the effects of ELS on autonomic and endocrine functioning, but also to examine the eligibility of these two indicators of stress reactivity in studies employing the MIST.

## 2 Methods

### 2.1 Participants

Thirty-two healthy participants were recruited via telephone and email from an existing database. Participants were screened for psychiatric disorders using the short version of the Structured Clinical Interview for DSM-IV (SKID; Wittchen et al., 1997). Inclusion criteria were absence of present or past diagnosis of neurologic or psychiatric disease, absence of major or unstable general medical conditions (diseases, illnesses, and injuries, e.g. neurologic disorders, heart conditions, stroke, pancreatic diseases, thyroid problems, cancer, infections) and fMRI eligibility. Accordingly, exclusion criteria were pregnancy, metal implants (e.g. ferromagnetic pacemakers, metal prostheses, aneurysm clips), head trauma or surgery, and claustrophobia. All participants gave written informed consent before participating and were reimbursed with 40 Euro. The study was carried out in accordance with the latest version of the Declaration of Helsinki and approved by the institutional review board of the Charité.

### 2.2 Study procedure

Pre-arrival instructions asked the participants to abstain from alcohol consumption and excessive exercise 24 h prior to the experiment, to abstain from any food or drink except for water and physical activity 1 h prior to the experiment as well as from smoking 30 min prior to the experiment, in order to avoid confounding of measurement (Task Force of the European Society of Cardiology and the North American Society of Pacing and Electrophysiology, 1996). To ensure relatively stable endogenous cortisol levels, all subjects arrived between 1 and 3 p.m. in our laboratory. Upon arrival the participants were told a cover story about a mental arithmetic task in order to measure concentration and arithmetic skills. Afterwards the fMRI scanning and saliva sampling procedures were explained and scanning started. Two saliva samples were taken before the Montreal Imaging Stress Task (MIST; Dedovic et al., 2005) began. Right after the MIST a third saliva sample was taken. After this, scanning was finished and the participants were debriefed about the true purpose behind the task. During the following 2 h the participants stayed in the laboratory to complete a set of questionnaires. Furthermore, additional saliva samples were taken. Subsequently participants were reimbursed for their participation. All in all, participation required a total of 3–4 h.

### 2.3 Measurements

#### 2.3.1 Early Life Stress

The Childhood Trauma Questionnaire (*CTQ*) was administered (Bernstein et al., 2003; Klinitzke et al., 2012). The *CTQ* consists of 28 items assessing five subscales of self-reported ELS: emotional neglect (*CTQ-EN*), emotional abuse (*CTQ-EA*), physical neglect (*CTQ-PN*), physical abuse (*CTQ-PA*), and sexual abuse (*CTQ-SA*). Items are answered on a 5-point Likert-scale ranging from 1 (*not at all*) to 5 (*very often*). Each subscale contains five items. Thus, scores on each subscale can range from 5 to 25 with higher values indicating higher exposure to ELS and a resulting total score (*CTQ-Total*) between 25 and 125. Additionally, the *CTQ* contains three items assessing the tendency to deny or minimize experienced childhood maltreatment. Depending on type of maltreatment effects can vary: Abuse tends to be associated with externalizing problems such as aggressive and delinquent behaviour whereas neglects tends to be associated with internalizing problems, e.g. social withdrawal and limited peer interactions (Arata et al., 2007; Hildyard and Wolfe, 2002). Thus, in addition to *CTQ-Total* discrete subscores were regarded as well. For some analyses, participants were grouped depending on severity of ELS. As participants in the present study did not report sexual abuse this discrete subscale was not regarded separately.

#### 2.3.2 Stress reactivity

Stress was induced using the Montreal Imaging Stress Task (MIST; Dedovic et al., 2005), which requires participants to perform mental arithmetic tasks. Three 7-min runs with three conditions were carried out. The first condition was a rest condition, in which no arithmetic task was shown. In the control condition the participant solved arithmetic tasks and immediately received feedback (“correct” vs. “incorrect”) via the screen. In the third condition, the stress condition, a time restriction was established. Feedback options were: “correct”, “incorrect”, and “time out”. Time limit and difficulty were automatically adjusted to be just beyond the individual's capacity. Furthermore, a coloured bar indicated the participant's performance compared to a (fictitious) norm sample's performance. Due to program design the participants' performance was always inferior to that of the norm sample. To further increase stress, the experimenter delivered a scripted negative verbal feedback after the first run and emphasized the need to improve performance via headphones. The experimenter requested the participants to repeat the task and increase their performance, indicating that due to under average performance the data would be useless otherwise. This personal negative feedback was given twice; after the first run and with greater emphasis after the second run. All three runs were carried out directly one after another, thus the MIST session took around 21 min in total. Participants were debriefed after the third run.

***Heart rate.*** A pulse oximeter (Siemens, model no. 07389567) attached on the index finger of the non-dominant hand was used to assess mean heart rate (HR) during the MIST (50 Hz sampling). Participants were requested to abstain from moving this hand during the measurement. After acquisition, R-peaks (maximum amplitude of the R wave in the QRS complex) were detected using Brain Vision Analyzer 2.1.1 (Brain Products GmbH). Inter-beat-intervals (IBIs) were computed as the time distance between two consecutive R-peaks. IBIs were later processed with Kubios HRV 2.1 (Tarvainen et al., 2014) and visually inspected for artifacts. Abnormal or biologically implausible data were excluded. HR is reported in beats per minute (bpm). The American Heart Association states the normal resting adult human heart rate is 60–100 bpm.

***Salivary cortisol level.*** Commercially available sampling devices (Sarstedt Inc., Rommelsdorf, Germany) were used to collect a total of five salivary cortisol samples. Cortisol level is reported in nanomoles per liter (nmol/L). The first two samples were collected 20 min prior to MIST onset (T_1_) and directly before the MIST (T_2_). The third sample was collected directly after the MIST (T_3_), before the participant was debriefed. The forth sample was collected 20 min after completing the MIST (T_4_), while the last sample followed 100 min after the MIST, directly before participants were dismissed (T_5_). Participants were instructed to move the cotton swab in a circular pattern for 1 min without actively chewing on it to ensure homogeneous saliva collection from all salivary glands. Saliva collection took place at room temperature, afterwards saliva samples were stored at −20 °C until biochemical analysis. For the analysis, saliva samples were thawed and spun at 2500 rpm for 5 min, yielding in a clear supernant. Cortisol concentration was determined in the neurobiology laboratory of the Department of Psychiatry, Charité - Universitätsmedizin Berlin. Free cortisol was analyzed using a commercially available TR-FRET-based (Fluorescence Resonance Energy Transfer) in-house adopted immunoassay (Cisbio International, Codolet, France). The analysis was carried out in accordance with the manufacturer's instructions. Intra-assay coefficients of variation were below 8%, inter-assay coefficients of variation were below 10%. Detection limits were 0.5 ng/ml for cortisol. Participants showing higher cortisol levels at T_4_ or T_3_ than at T_2_ were defined as responders, the rest as non-responders (Pruessner et al., 2008; Schwabe et al., 2008).

### 2.4 Statistical analyses

Descriptive statistics for sociodemographic data and study variables were examined. T-tests were used to check for gender differences. ANOVAs for repeated measures were used to analyse HR responses during MIST. First of all, main effects of MIST condition (rest, control, and stress) and MIST runs (3) as within-subjects factors on HR were explored. ANOVAs for repeated measures were also used for examining changes in salivary cortisol level over the five sampling points. Correlations between HR and salivary cortisol levels were computed. Furthermore, to examine whether HR differed for cortisol responders and non-responders, an ANOVA for repeated measures was computed with HR as within-subject factor and responders vs. non-responder as between-subject factor.

For examining the relationship between ELS and stress reactivity, ELS exposure of participants was classified into three categories of severity: no, moderate, and severe. Severity of *CTQ*-Total and the four *CTQ* subscores was categorized separately, based on the corresponding percentiles. Participants with a score ≤33rd percentile were assigned to the no ELS category, participants with a score ≤66th percentile to the moderate ELS category and participants with a score >66th percentile were assigned to the severe ELS category. Thus for *CTQ*-Total and each subscale a distinct 3-level categorization resulted. Subsequently, ANOVAs for repeated measures were performed with HR in the MIST conditions (rest, control, and stress) and MIST runs (3) as within-subjects factors and ELS categorization for *CTQ*-Total, *CTQ*-EN, *CTQ*-EA, *CTQ*-PN, and *CTQ*-PA separately as between-subject factors. Same for examining the effects of ELS on salivary cortisol level: ANOVAs for repeated measures were performed with salivary cortisol level at the five sampling points as within-subjects factor and the ELS categorization for *CTQ-Total* and the subscores as between-subjects factor, separately. T-tests for independent sampling were used to examine whether amount of ELS differed for cortisol responders vs. non-responders. All tests were two-tailed and the significance threshold was set at *p* < .05. All results were corrected by the Greenhouse-Geisser procedure where appropriate (violation of sphericity assumption). Statistical analyses were performed using SPSS (IBM SPSS Statistics for Macintosh, Version 20.0. Armonk, NY: IBM Corp.).

## 3 Results

### 3.1 Sample characteristics

Twelve subjects had to be excluded from the sample due to missing data or insufficient quality of the HR-data. For the remaining subjects (N = 20) means and standard deviations of sociodemographic data and study variables are summarized in Table 1. Sociodemographic data and study variables were not intercorrelated except for age and emotional abuse (*r* = 0.45, *p* = .03), age and physical abuse (*r* = 0.48, *p* = .03) and age and intelligence (*r* = 0.60, *p* = .01). No gender differences were found (all tests *p* > .05).

**Table 1:** Means and standard deviations for sociodemographic data and study variables.

	*M*	*SD*
Gender		
Male, *n*	11	
Female, *n*	9	
Age	36.30	11.63
IQ	115.15	15.46
Years of Education	13.80	2.44
*CTQ-Total*	35.80	12.83
*CTQ-EN*	9.80	5.54
*CTQ-EA*	7.75	3.57
*CTQ-PN*	6.80	2.71
*CTQ-PA*	6.40	2.93
HR	78.46 bpm	13.32 bpm
Cortisol Level	1.30 nmol/l	.66 nmol/l

*Note. N* = 20. *M* = mean; *SD* = standard deviation; *IQ* = intelligence quotient; *CTQ-Total* = Childhood Trauma Questionnaire Total score; CTQ-EN = Childhood Trauma Questionnaire emotional neglect subscale; *CTQ-EA* = Childhood Trauma Questionnaire emotional abuse subscale; *CTQ-PN* = Childhood Trauma Questionnaire physical neglect subscale; *CTQ-PA* = Childhood Trauma Questionnaire physical abuse subscale; HR = heart rate. Heart rate is reported in beats per minute (bpm). Cortisol level is reported in nanomoles per liter (nmol/l).

### 3.2 Stress reactivity analyses

**Fig. 1:**
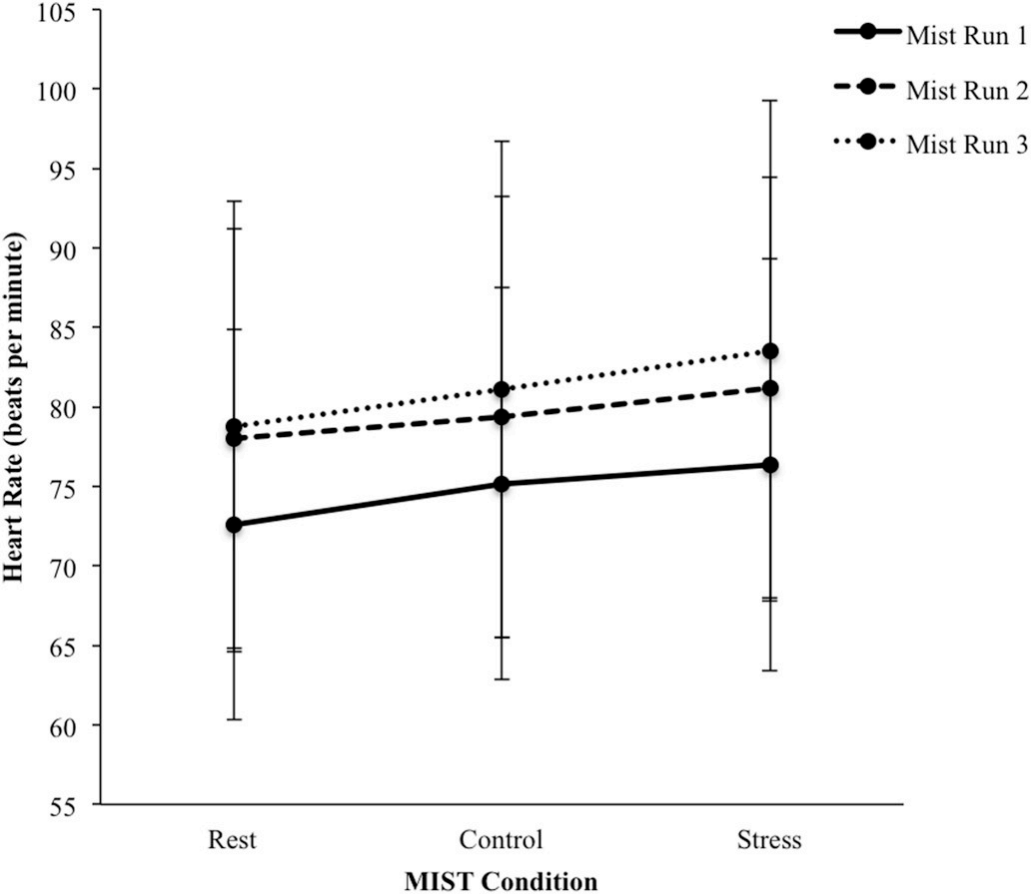
Mean HR levels for all three MIST runs during rest, control, and stress condition. Error bars represent standard deviations. MIST = Montreal Imaging Stress Task.

**HR.** First of all, main effects of MIST condition (rest, control, and stress) and MIST runs (3) as within-subjects factors on HR were examined. ANOVA for repeated measures revealed a highly significant main effect of MIST conditions on HR, *F*(2, 38) = 25.83, *p* = .00, *η*^*2*^ = 0.58. Post hoc analyses revealed significant differences between HR in all conditions (all tests *p* < .01). HR was highest in the stress condition (*M* = 80.37, *SE* = 3.06) and lowest in the rest condition (*M* = 76.46, *SD* = 12.88). There was also a highly significant main effect of MIST runs on HR, *F*(2, 38) = 17.23, *p* = .00, *η*^*2*^ = 0.48. Post hoc analyses revealed significant HR differences between all runs (*p* < .05). HR was highest in the third run (*M* = 81.14, *SD* = 15.07) and lowest in the first run (*M* = 74.72, *SD* = 12.39). No condition × run interaction effect was found. See Fig. 1 for graphical display.

**Salivary Cortisol.** ANOVA for repeated measures with salivary cortisol level at the five sampling points as within-subjects factor was used to test whether cortisol levels variied significantly across the five measurement points and revealed a significant effect *F*(4,76) = 8.88, *p* = .00, *η*^*2*^ = 0.32. Post hoc tests revealed significant differences between T_1_ and T_2_, T_1_ and T_3_, T_1_ and T_5_, T_2_ and T_5_, T_3_ and T_5_ and T_4_ and T_5_ (*p* < .05). Thus, cortisol level was highest before the MIST, and lowest at the end of the experiment. During and 20 min after the MIST no significant change of salivary cortisol was found.

Participants were divided into cortisol responders and non-responders as described above. Each group consisted of *n* = 10 participants. ANOVA for repeated measures with salivary cortisol level at the five sampling points as within-subjects factor and responder vs. non-responder as between-subjects factor did not reveal a significant difference between responders vs. non-responders. Yet, a sampling point x responder vs. non-responder interaction was found *F*(4,72) = 2.53, *p* = .05, *η*^*2*^ = 0.12.

**Fig. 2:**
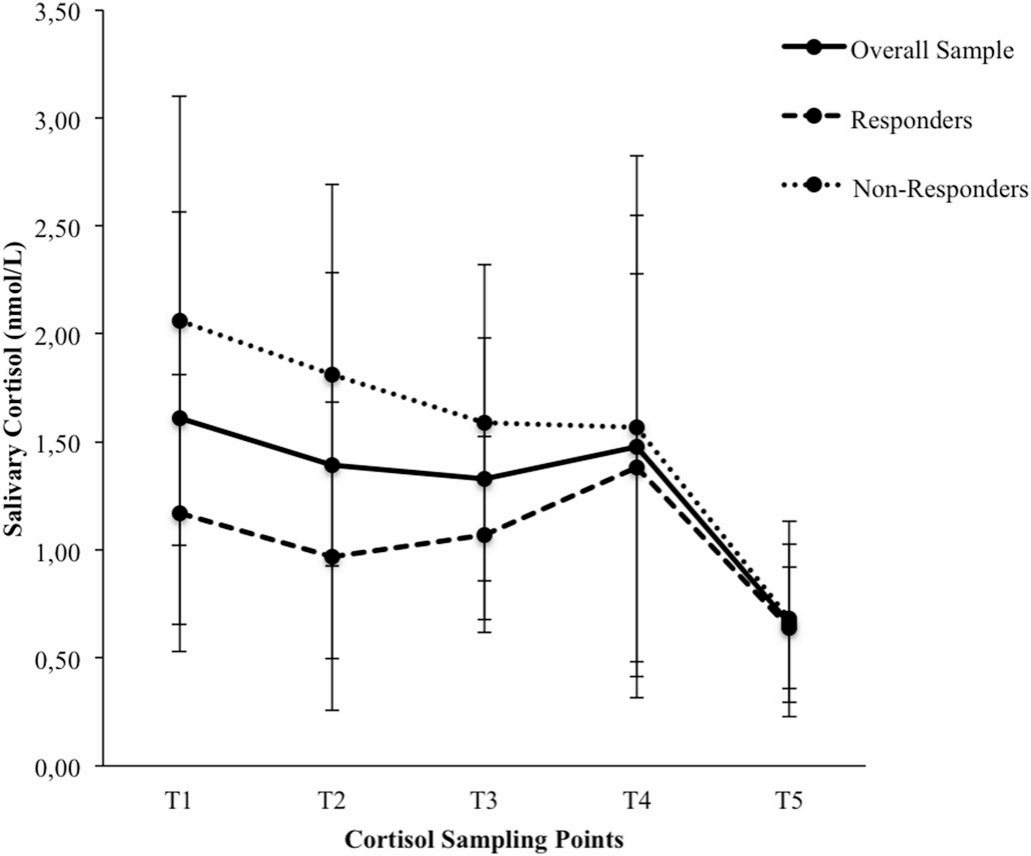
Salivary cortisol levels at the five sampling points for overall sample, responders and non-responders. Error bars represent standard deviations. nmol/L = nanomoles per liter.

Post-hoc tests revealed significant differences between the two groups at T_1_ and T_2_ *T*(18) = 2.30, *p* = .03, *η*^*2*^ = 0.23; *T*(18) = 2.34, *p* = .03, *η*^*2*^ = 0.23, respectively. For graphical display see Fig. 2. Responder differed significantly between T_3_ and T_5_ (*p* < .05), non-responder between T_1_ and T_5_, T_2_ and T_5_ and T_3_ and T_5_ (*p* < .05). Thus, non-responder show a significantly higher cortisol level at the beginning of the experiment than responder. Yet, in neither group the changes in cortisol level during and 20 min after the MIST were significant.

**HR and Salivary Cortisol.** No correlation between HR and salivary cortisol level was found (all *p* > .05). ANOVA for repeated measures did not reveal a significant effect of cortisol level as between-subject factor: HR did not differ significantly between cortisol responders and non-responders in any run or any condition of the MIST.

**ELS and HR**. Categorizations of ELS were used to examine the relationship between ELS and HR. Demographics did not differ significantly between ELS categories, except for a significant IQ difference between the *CTQ-PN* categories. Participants in the severe *CTQ-PN* category had a significantly higher IQ than participants in the no *CTQ-PN* category *F*(2,17) = 8.72, *p* = .002. Furthermore there was a significant age and IQ difference between *CTQ-Total* categories. Participants in the severe *CTQ-Total* category were significantly older, *F*(2, 17) = 4.32, *p* = .03 than participants in the moderate *CTQ-Total* category, and had a significantly higher IQ *F*(2, 17) = 4.55, *p* = .03 than participants in the no *CTQ-Total* category. Thus, age and IQ were added as covariates for these analyses.

There was no significant main effect for *CTQ-Total* on HR. Numerically, however, HR was lowest in the severe ELS category (*M* = 71.98, *SD* = 24.64) and higher in the no ELS and moderate ELS categories (*M* = 76.28, *SD* = 22.67; *M* = 88.55, *SD* = 24.10, respectively), for all MIST runs and conditions. No *CTQ-Total* x condition interaction was found, neither was a *CTQ-Total* x run interaction.

The three-way interaction *CTQ-Total* x condition x run was also not significant. Accordingly, no significant main or interaction effects for any of the *CTQ* subscales on HR were found, however, as for *CTQ-Total*, for all *CTQ* subscales HR was lowest in the severe ELS category.

**ELS and Salivary Cortisol.** Categorizations of ELS were used to examine the relationship between ELS and salivary cortisol level as well. There was no significant main effect for *CTQ-Total* on salivary cortisol level. Numerically, cortisol level was lowest in the severe ELS category (*M* = 1.08, *SD* = 1.12) and higher in the no ELS and moderate ELS categories (*M* = 1.24, *SD* = 1.12; *M* = 1.62, *SD* = 1.21, respectively), for all timepoints of measurement. No *CTQ-Total* x cortisol sampling points interaction was found. Accordingly, no significant main or interaction effects for any of the *CTQ* subscales on salivary cortisol level were found. No significant correlation between ELS and salivary cortisol level was found (all *p* > .05). Cortisol responders and non-responders did not differ significantly concerning the severity or type of reported ELS (all *p* > .05).

## 4 Discussion

This study aimed to gain a better understanding of the effect of ELS on HR and salivary cortisol level as both autonomic and endocrine indicators of individual stress reactivity. Stress was induced with the MIST. Moreover, we also aimed to examine the general eligibility of HR and salivary cortisol as outcome variables for research employing the MIST.

While there was no significant effect of ELS on HR, it was lowest in the severe ELS category regardless of MIST condition and run. This is in accordance with findings from Carpenter et al. (2007), who examined a non-clinical community sample and found evidence for diminished stress reactivity in participants with ELS. Considering the absence of significant effects in the current study, the severity and type of ELS in the present healthy sample needs to be regarded. Also, we chose a percentile oriented approach to categorize ELS severity, since we investigated healthy subjects that represent the middle range of socioeconomic status in the general population of Germany and are therefore much less impoverished than subjects in previous studies investigating effects of ELS. As a consequence, subjects experienced overall less exposure to ELS and less ELS severity, thus our approach might have overestimated the severity of ELS in some participants. Reduced stress reactivity in participants with moderate ELS would be in accordance with *biological sensitivity to context*, a concept proposed to integrate the seemingly contradictory findings concerning ELS and stress reactivity (Boyce and Ellis, 2005). It proposes a u-shaped curvilinear relationship between exposure to early adversities and stress reactivity. Thus high-reactive phenotypes emerge from highly protected as well as highly stressful early environments. Under highly adverse circumstances it may be crucial for survival to be very aware of potential threats and to react promptly, thus in those children stress reactivity is up-regulated. Stress reactivity is also up-regulated in children experiencing extremely supportive conditions, allowing a greater receptiveness to social resources. It should not be ignored though, that the majority of children is growing up under neither extremely supportive nor highly threatening circumstances and therefore adequate stress reactivity is maintained.

We further investigated salivary cortisol as an endocrine indicator of individual stress reactivity. Similar to HR, no significant effect of ELS on salivary cortisol level was found, yet it was lowest in the severe ELS category regardless of timepoint of measurement. Considering that participants in the severe ELS category experienced rather moderate ELS regarding to the whole range of ELS, these findings for salivary cortisol would be in accordance with *biological sensitivity to context*, as for HR. A possible explanation for the absence of significant effects might be, as mentioned above, the small range and type of ELS in the present sample. For example, previous findings include participants who experienced much more severe stressors such as loss of a parent at early age or growing up in an orphanage (Heim and Nemeroff, 2002). Moreover, previous studies often included participants with a history of sexual abuse while our participants did not report sexual abuse (Elzinga et al., 2008; Heim and Nemeroff, 2002). Furthermore, the differing findings might also be due to differences in the assessment of ELS as previous studies did not necessarily use the CTQ but rather the Traumatic Experiences Check List (TEC, Nijenhuis et al., 2002) or the Early Trauma Inventory (ETI, Bremner et al., 2000).

Previous studies employing the MIST used salivary cortisol as an indicator for the successful induction of psychosocial stress (Dedovic et al., 2005). The present findings, however, suggests that HR might be much better suited, since unlike salivary cortisol it allows for the differentiation between the MIST conditions and runs. HR differed significantly between all MIST conditions and runs and was highest in the stress condition and lowest in the rest condition. Also, HR increased significantly during the three runs. On the other hand, results of the cortisol analyses appeared to be much less specific. In accordance with previous studies (Dedovic et al., 2005) several saliva samples were taken prior and after the MIST in order to measure cortisol levels. Even though salivary cortisol levels varied significantly over the five sampling points, they were highest at the beginning of the experiment as well as 20 min before MIST onset and lowest at the end of the experiment, 100 min after the MIST finished. Therefore, in contrast to HR, the MIST induced no significant changes in salivary cortisol levels.

When participants were divided into cortisol responders and non-responders (Pruessner et al., 2008), only 50% of the sample could be considered as responders, which is, however, in accordance with previous studies. Responders and non-responders did not differ concerning the amount of reported ELS. Interestingly, the two groups only differed significantly concerning their cortisol level at the first two sampling points, before MIST onset, which is contrary to previous results (e.g. Dedovic et al., 2009). Non-responders showed significantly higher cortisol levels at T_1_ and T_2_ than responders, whereas no differences were found for the following sampling points. As a chronically altered activity of the HPA-axis can be associated with several physical and psychological impairments, such as type 2 diabetes and depression (Carpenter et al., 2009), it remains very important to further examine how these differences in salivary cortisol levels emerge. It is important to note though, that for neither of the groups the MIST induced a significant change in salivary cortisol level. Overall, our findings show no good fit of cortisol data and MIST structure, while HR seems to be an extremely sensitive measure to depict the varying runs and conditions of the MIST. Thus, for studies employing the MIST, HR seems to be the better fitting outcome variable.

We found no association between HR and salivary cortisol level. As this is in accordance with previous findings (e.g. Kirschbaum et al., 1993), it underlines the importance of assessing multiple indicators of stress reactivity. However, for examining effects on cortisol other tests such as a socially evaluated cold-pressor test (Schwabe et al., 2008) or the combined dexamethasone/CRH test (Zobel et al., 2001) might be better suited than the MIST.

In general, cortisol data are more prone to confounds, e.g. through time of the time of day, eating, smoking, gender, or menstrual cycle (Dickerson and Kemeny, 2004; Kirschbaum et al., 1993). Moreover, there is a great variation considering the analysis and interpretation of cortisol data (responder vs. non-responder, baseline to peak interval, area under the curve), which impedes obtaining comparable results. These circumstances should be considered in future research.

A few limitations of the present study need to be mentioned. First of all, the sample was rather small with a limited range of ELS experience. As ELS prevalence and severity is higher in clinical than in non-clinical samples (Bernstein et al., 2003), subsequent studies with a mixed clinical and non-clinical sample might be insightful. Even though the *CTQ* is a thoroughly evaluated instrument (Bernstein et al., 2003), longitudinal studies might be helpful to avoid confoundation by recall bias. Moreover, assessing age at onset as well as frequency and chronicity of ELS seems to be advisable (Heim and Nemeroff, 2001). Considering the assessment of HR it needs to be mentioned that general fitness was not assessed. As general fitness influences cardiovascular reactivity, confounding influences cannot be excluded (Huang et al., 2013). Additional assessment of perceived stressfulness of the stress task might prove enlightening, e.g. with a Visual Analog Scale.

## 5 Conclusions

In conclusion, the current study did not detect effects of mild to moderate ELS on HR and salivary cortisol level as two distinct indicators of individual stress reactivity. However, this result should not lead to a general underestimation of the impact of ELS on stress reactivity, as our sample comprised only healthy subjects without severe ELS experiences. Future studies with larger clinical samples covering a wider range of ELS experiences might shed some more light on the effects of ELS on distinct measures of stress reactivity. Importantly, our study showed that heart rate is a more feasible indicator of an individual's stress reactivity than salivary cortisol. Future studies should therefore not solely rely on salivary cortisol as an indicator of stress reactivity, as this might lead to an oversight of more subtle effects of psychosocial stress.

## Ethics approval and consent to participate

All participants gave written informed consent before participating and were reimbursed with 40 Euro. The study was carried out in accordance with the latest version of the Declaration of Helsinki and approved by the institutional review board of the Charité.

## Conflicts of interest

The authors declare that they have no competing interests.

## Funding

The study was supported by the German Research Foundation (GR 4510/2-1).

## Figures and Tables

**Figure:**
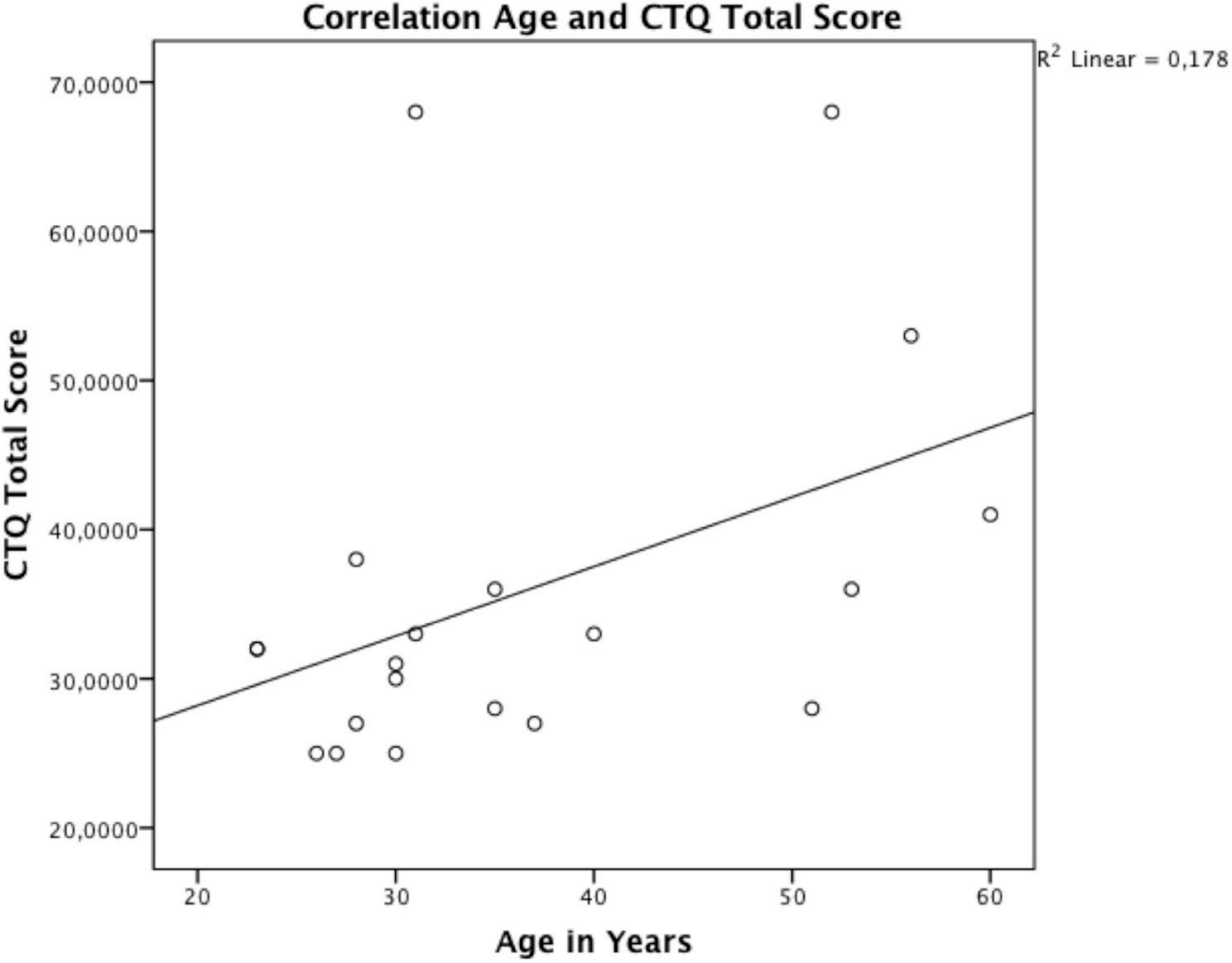
Scatterplot 1: Correlation between Age and CTQ Total Score.

**Figure:**
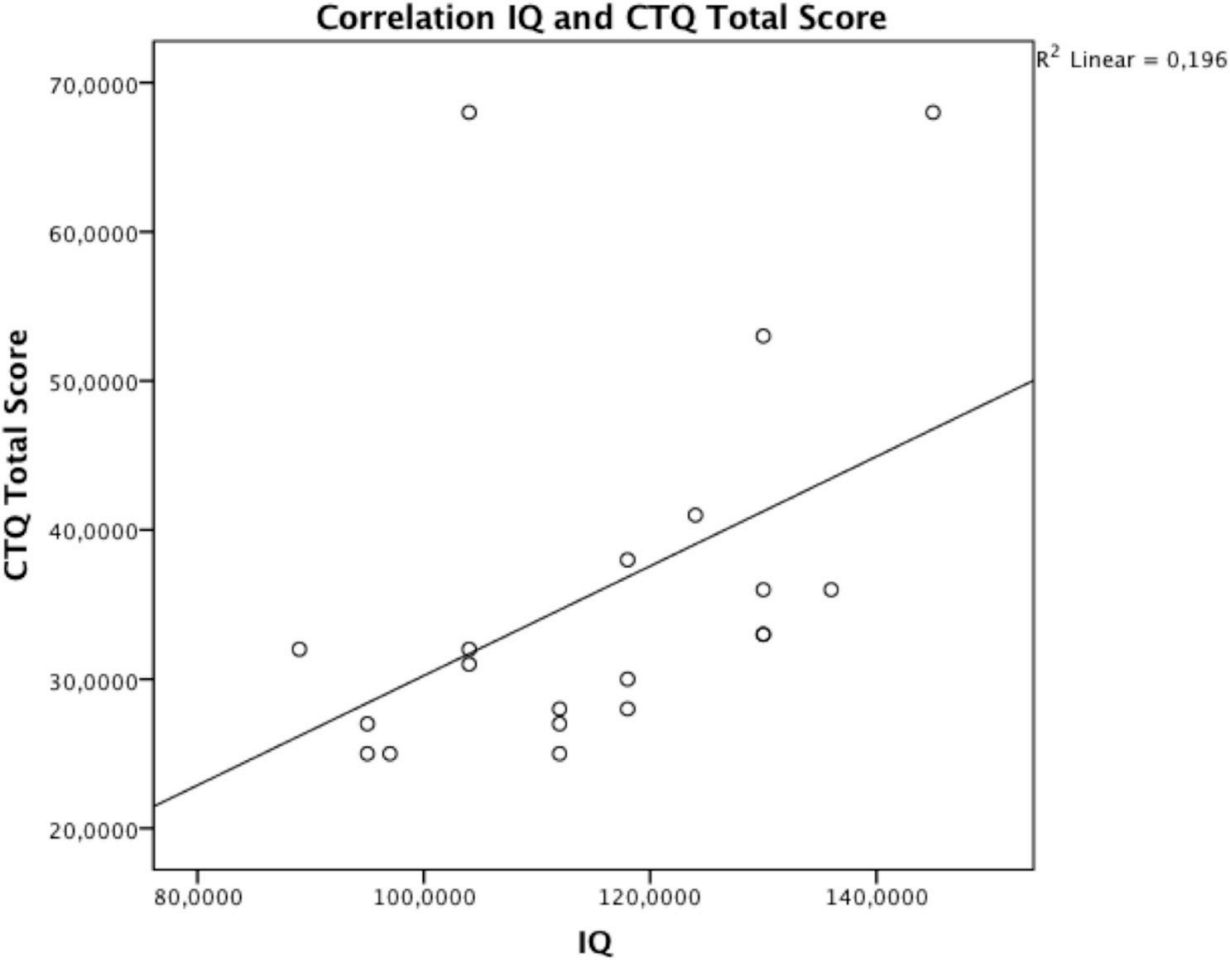
Scatterplot 2: Correlation between IQ and CTQ Total Score.

**Figure:**
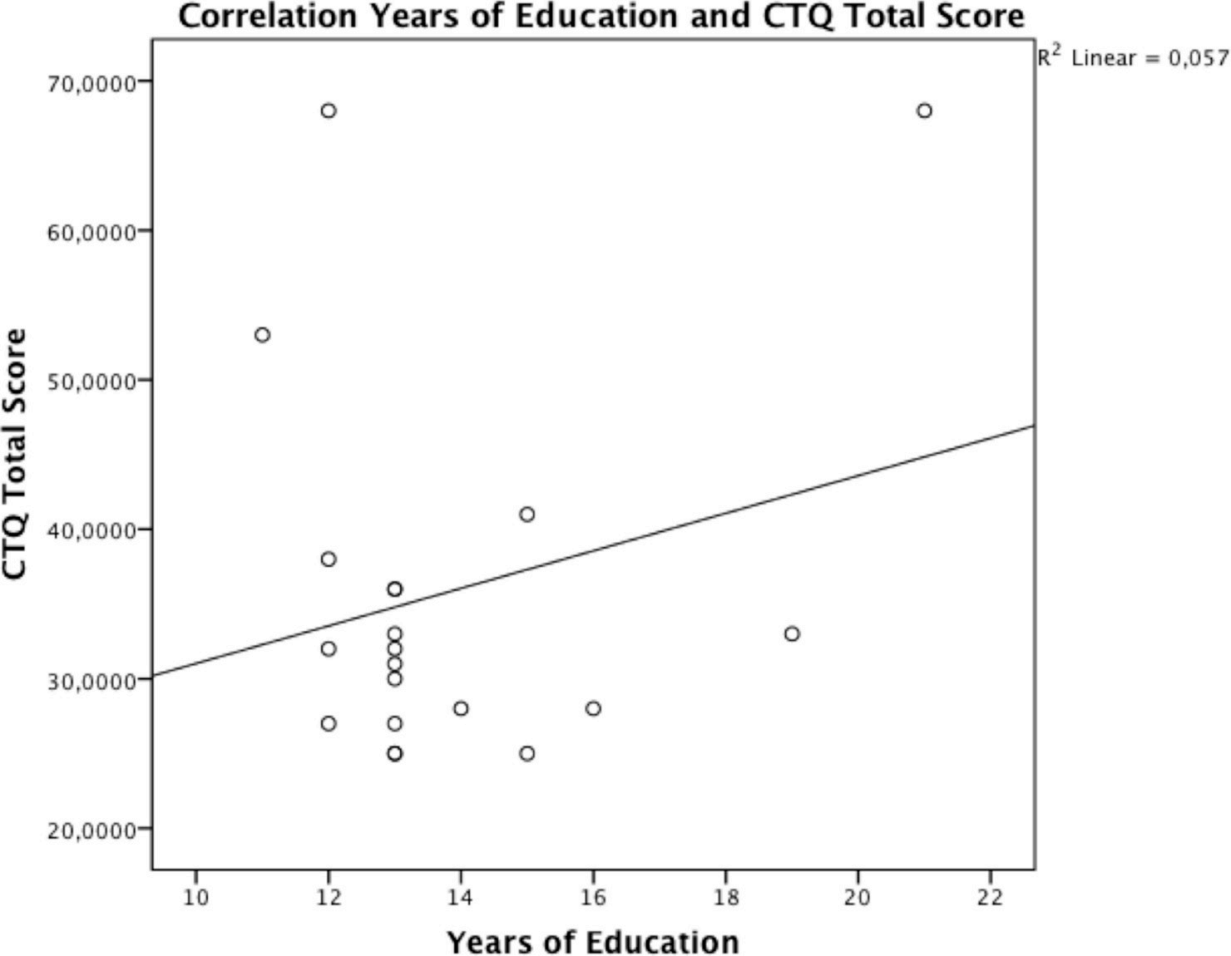
Scatterplot 3: Correlation between Years of Education and CTQ Total Score.

## Appendix ASupplementary data

Data Profile Data Profile
